# Alternative fatty acid desaturation pathways revealed by deep profiling of total fatty acids in RAW 264.7 cell line

**DOI:** 10.1016/j.jlr.2023.100410

**Published:** 2023-07-10

**Authors:** Tian Xia, Xue Jin, Donghui Zhang, Jitong Wang, Ruijun Jian, Hang Yin, Yu Xia

**Affiliations:** 1 MOE Key Laboratory of Bioorganic Phosphorus Chemistry & Chemical Biology, Department of Chemistry, Tsinghua University, Beijing, China; 2 School of Pharmaceutical Sciences, Tsinghua University, Beijing, China; 3 State Key Laboratory of Precision Measurement Technology and Instruments, Department of Precision Instrument, Tsinghua University, Beijing, China; 4 Tsinghua-Peking Center for Life Sciences, Tsinghua University, Beijing, China; 5 Beijing Frontier Research Center for Biological Structure, Tsinghua University, Beijing, China

**Keywords:** Unsaturated fatty acids, double bond location isomers, mass spectrometry, Paternò–Büchi reaction, RAW 264.7 macrophage

## Abstract

In-depth structural characterization of lipids provides a new means to investigate lipid metabolism. In this study, we have conducted deep profiling of total fatty acids (FAs) from RAW 264.7 macrophages by utilizing charge-tagging Paternò-Büchi derivatization of carbon-carbon double bond (C=C) and reversed-phase liquid chromatography-tandem mass spectrometry. A series of FAs exhibiting unusual site(s) of unsaturation was unearthed, with their identities being confirmed by observing anticipated compositional alterations upon desaturase inhibition. The data reveal that FADS2 Δ 6-desaturation can generate n-11 C=C in the odd-chain monounsaturated fatty acids (MUFAs) as well as n-10 and n-12 families of even-chain MUFAs. SCD1 Δ 9-desaturation yields n-6, n-8, and n-10 of odd-chain MUFAs, as well as n-5, n-7, and n-9 families of even-chain MUFAs. Besides n-3 and n-6 families of polyunsaturated fatty acids (PUFAs), the presence of n-7 and n-9 families of PUFAs indicates that the n-7 and n-9 isomers of FA 18:1 can be utilized as substrates for further desaturation and elongation. The n-7 and n-9 families of PUFAs identified in RAW 264.7 macrophages are noteworthy because their C=C modifications are achieved exclusively via de novo lipogenesis. Our discovery outlines the metabolic plasticity in fatty acid desaturation which constitutes an unexplored rewiring in RAW264.7 macrophages.

Fatty acid (FA) metabolism consists of anabolic and catabolic processes that are necessary for energy homeostasis as well as the formation of metabolic intermediates required for the maintenance of cell membrane structure and function (1), energy storage (2), and cell signaling (3). The desaturases introduce a carbon–carbon double bond (C=C) at a specific position on the acyl chain, thereby influencing several key biological properties of the fatty acids, including membrane fluidity (4), antioxidant activity (5), and inflammation (6). Mammalian cells express desaturases of Δ 5, Δ 6, and Δ 9 activities, where the Δ− number indicates the position of a C=C counting from the carboxylic acid moiety of FA. The desaturases are classified into two distinct families referred to as stearoyl-coenzyme A desaturase (SCD) (7, 8) and fatty acid desaturase (FADS) (9). Cancer cells have an aberrantly activated lipid metabolism that enables them to synthesize, elongate, and desaturate fatty acids to support proliferation (10). Notably, it was recently shown that cancer cells exploit FADS2 to catalyze n-10 C=C formation (i.e., the 10^th^ carbon counting from the FA methyl terminus) in palmitic acid (FA 16:0) (11), which is usually observed only within lipids from hair and skin (12); however, this desaturation mechanism can be activated to meet the metabolic needs of cancer. Young *et al.* revealed the promiscuity of human FADS2, which promoted the production of rarely reported n-8, n-10, and n-12 desaturation sites in lipids (13). The studies from the Brenna group showed that FADS2 activity was promiscuous. It can catalyze Δ4, Δ6, and Δ8 desaturation towards at least 16 substrates, including saturated fatty acids, even- and odd-chain monounsaturated fatty acids (MUFAs), polyunsaturated fatty acids (PUFAs), and branched-chain fatty acids (BCFAs) with chain length ranging from 16 to 24 carbons (14, 15). In order to elucidate unexplored plasticity in cancer cells fatty acid metabolism, it is imperative to identify the unsaturation profile of the fatty acyl building blocks. Gas chromatography hyphenated with electron ionization mass spectrometry (GC/EI-MS) is still widely used for profiling of FAs, albeit in the form of fatty acid methyl esters (FAMEs) (16, 17, 18). Identification of FAs relies on matching the retention time of GC-separated FAMEs to those of the standards (16, 17, 18), which inevitably hinders its utility in the discovery of unknown FAs. To overcome this limitation, the Brenna group combined acetonitrile (ACN) chemical ionization (CI) with collision-induced dissociation (CID) for independent structural analysis of unsaturated FAMEs (19). Alternatively, new tandem mass spectrometry (MS/MS) methods, such as ozone-induced dissociation (OzID) (20), ultraviolet photodissociation (UVPD) (21, 22), epoxidation (23, 24), aziridination (25, 26), and the Paternò–Büchi (PB) derivatization (27, 28, 29, 30, 31), have been developed to provide lipidome profiling at the level of C=C positions. For instance, our group recently developed a sensitive and readily adaptable workflow for the quantitation of FAs at C=C location level via charge-tagging PB derivatization and reversed-phase liquid chromatography-tandem mass spectrometry (RPLC-MS/MS) (31). This method has advantages in identifying unknown FAs where synthetic standards are not available. It offers a limit of detection (LOD) in the sub-nM range for synthetic standards and relative quantitation of low abundance C=C location isomers (as lows as 1% relative to the most abundant isomer).

In this work, we employ charge-tagging PB derivatization in combination with desaturate inhibition to uncover a diverse set of novel fatty acids with an unusual site(s) of unsaturation in RAW 264.7 macrophages. The latter method allows us to achieve high confidence in the identification even in the absence of authentic standards. Furthermore, desaturate inhibition provides evidence for studying FAs derived from SCD1 Δ9-desaturation and FADS2 Δ6-desaturation activities. Our data suggest that both enzymes display broad activity toward both even and odd-chain saturated FAs and give the cells access to an expanded repertoire of even-chain MUFAs with n-5, n-7, n-9, n-10, and n-12 C=Cs. Furthermore, even-chain MUFAs derived from these de novo synthesized families also act as substrates for further desaturation and elongation, yielding a wide array of PUFAs, consisting of the n-7, n-9, n-10, and n-12 families.

## Materials and Methods

### Lipid nomenclature

The shorthand notation recommended by LIPID MAPS consortium was used (32). In brief, the position of C=C in an aliphatic chain is defined by the *n-x* nomenclature, counting from the methyl terminus. The Z/E stereo-configurations of C=C could not be assigned from PB-MS/MS and thus were not indicated for FAs from biological samples.

### Chemicals and biological samples

High-performance LC (HPLC) grade iso-propanol (IPA), acetonitrile (ACN), methanol (MeOH), and water were purchased from Fisher Scientific. Methyl tert-butyl ether (MTBE), formic acid (HCOOH), ammonium formate, and 2-acetylpyridine (2-acpy) were purchased from Sigma Aldrich. Lipid standards were purchased from Avanti Polar Lipids. AMP+ MaxSpec Kit was purchased from Cayman Chemical.

RAW 264.7 macrophages (American Type Culture Collection (ATCC); Manassas, VA, USA) were cultured in Roswell Park Memorial Institute (RPMI) 1640 Medium with 10% fetal bovine serum and 1% Penicillin-Streptomycin solution and collected by centrifugation. The cell pellets were washed, frozen in liquid nitrogen, and stored at −80°C. For direct inhibition of enzyme activity, RAW 264.7 macrophages were treated for 72 h with 60 μM FADS2 inhibitor (SC26196, purchased from Sigma-Aldrich) and 2.5 μM SCD1 inhibitor (CAY10566, purchased from Sigma-Aldrich), while the control group was treated with DMSO.

### Lipid extraction and saponification

The cell samples were extracted using a modified MTBE protocol (33). In brief, MTBE (5 ml), MeOH (1.5 ml), water (1.25 ml), and 1 mM [D4] FA 18:0 (10 μl) were added to the tube containing 2 million cells. The mixture was then vortexed for 5 min. To separate the organic and aqueous phases, the mixture was centrifuged at 10,000 *g* for 10 min. The upper phase was collected. The lower phase was re-extracted with 2 ml of the upper phase of the system MTBE/MeOH/H_2_O (10/3/2.5, v/v/v). Finally, the combined organic phases were collected and dried under N_2_ flow for further use. The cell medium was also subjected to lipid extraction and total FA profiling. The extracted lipids were saponified in 500 μl ACN:15% KOH (50/50, v/v) at 60°C for 60 min. The solution was acidified with 1M HCl (1 ml). The hydrolyzed total FAs were extracted twice with 1.5 ml isooctane each time. The organic layer was collected, dried under nitrogen, and redissolved in an aliquot of 500 μl MeOH for further derivatization.

### AMPP derivatization

AMPP derivatization followed the procedure provided by the vendor (AMP+ MaxSpec Kit). AMPP derivatized sample was extracted twice by 600 μl MTBE and dried under a nitrogen stream. The derivatized sample was resuspended in 100 μl MeOH for RPLC-MS/MS analysis.

### Offline 2-acpy PB derivatization of total FAs

The 2-acpy PB derivatization was performed using a home-made flow microreactor (34). Total FA extracts and 10 mM 2-acpy were dissolved in 200 μl ACN. The solution was injected into the flow microreactor for 20 s’ UV-irradiation (∼254 nm). About 200 μl reaction solution was collected; The PB derivatized sample was subjected to subsequent RPLC-MS/MS analyses.

### RPLC-MS/MS

RPLC-MS/MS analyses were conducted on an Acquity UPLC I-Class system (Waters) hyphenated with a hybrid trapped ion mobility-quadrupole time-of-flight mass spectrometer (timsTOF, Bruker Daltonics, Bremen, Germany). A CORTECS UPLC C18 column (150 mm × 2.1 mm, 1.6 μm, Waters, Milford, MA, USA) was used for separation. The mobile phase A contained H_2_O:ACN (40:60, v:v, added with 10 mM ammonium formate) and mobile phase B contained IPA:ACN (40:60, v:v, added with 0.1% HCOOH). The flow rate was set at 0.5 ml/min. Oven temperature was set at 60°C. The chromatographic gradient was as follows: 30% B at 0–0.4 min, 30%–45% B at 0.4–0.9 min, 45%–52% B at 0.9–1.1 min, 52%–58% B at 1.1–1.8 min, 58%–66% B at 1.8–2.5 min, 66%–70% B at 2.5–3.1 min, 70%–75% B at 3.1–4 min, 75%–97% B at 4–4.5 min, 97% B at 4.5–6 min, 30% B at 6.1–7 min. The MS parameters were optimized as follows: capillary voltage, 4500 V; end plate voltage, 500 V; nebulizer, 3 Bar; dry gas, 10 L/min; dry temperature, 210°C; CID energy for MS/MS, 22 eV.

### Data analysis and lipid identification

The analysis of RPLC-PB-MS/MS data was carried out using a home-made software, LipidNovelist (35). Data Analysis 5.0 software (Bruker Daltonics) was used to convert .d data to an open file format (.ascii), which can be read by LipidNovelist. LipidNovelist conducted de novo analysis of the data generated from RPLC-PB-MS/MS and the C=C location of each unsaturated fatty acid was assigned based on the detection of C=C diagnostic ion pairs. LipidNovelist also calculated the intensity ratios of diagnostic ions corresponding to C=C location isomers for relative isomer quantitation. For relative quantitation at the sum composition level, MS^1^ spectra of AMPP-derivatized fatty acid and corresponding internal standards were imported into the LipidNovelist Extension and subjected to Type-I isotope correction. The most up-to-date version of LipidNovelist and LipidNovelist Extension, along with instructional videos and example data to facilitate its utilization, can be accessed at https://doi.org/10.6084/m9.figshare.22297771.

## Results

### Analysis workflow

**Fig. 1:**
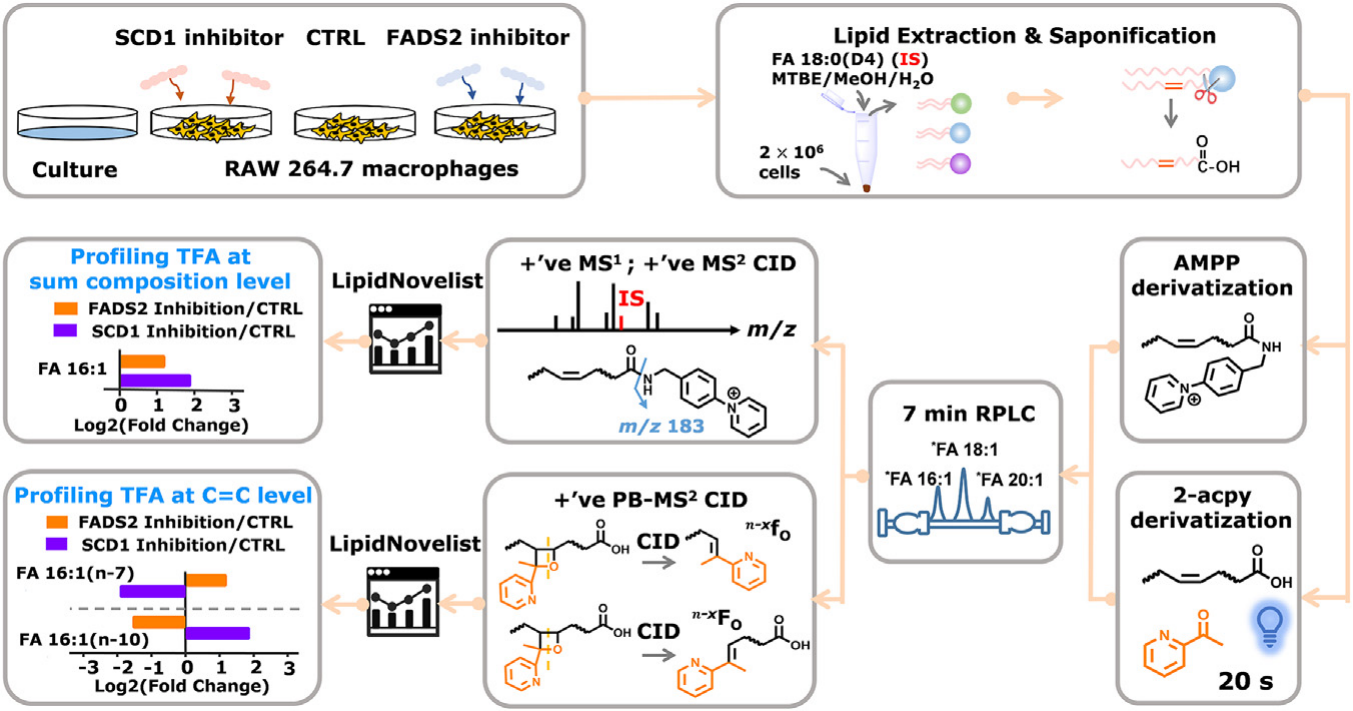
Analytical workflow for deep profiling of total FAs in RAW 264.7 macrophages and culture medium. The workflow includes cell culturing with and without desaturase inhibition, lipid extraction, saponification, 2-acetylpyridine (2-acpy) derivatization, AMPP derivatization, and subsequent analysis using reversed-phase liquid chromatography-tandem mass spectrometry (RPLC-MS/MS) for relative quantitation of total FAs at both the sum composition and C=C location levels.

RAW 264.7 cells have been extensively characterized and are well-suited for lipid metabolism studies due to their expression of a wide range of fatty acid desaturation enzymes involved in lipid metabolism (36, 37, 38). Figure 1 provides an overview of the analysis workflow. After lipid extraction and saponification, [D4] FA 18:0 was added as the internal standard (IS). The total FAs were divided into two equal aliquots. One aliquot underwent AMPP derivatization, while the other aliquot was subjected to 2-acpy derivatization. Subsequently, the AMPP-derivatized FAs were subjected to RPLC-MS analysis. LipidNovelist Extension was used to process the MS^1^ data which provided relative quantification (I/I_IS_) at the sum composition level (35). The %relative abundance of a certain FA was calculated by normalizing its MS^1^ signal to that of all FAs detected in the same RPLC run. Type-I isotope correction was applied to the data prior to relative quanaitation. The associate data are provided in supplemental Table S1.

Then, we used 2-acpy derivatization (the PB reaction) followed by RPLC-MS/MS to achieve relative quantitation of unsaturated total FAs at the C=C location level (31). The PB-MS/MS data were analyzed by the LipidOA module (39) embedded in LipidNovelist (35) to achieve de novo identification of FA at the C=C location level. %Relative composition of a specific isomer was calculated by normalizing the relative abundances of the C=C diagnostic ions associated with that isomer to the summed abundances of the diagnostic ions from all C=C location isomers. In order to enhance the accuracy of identification for uncanonical FA C=C location isomers especially in the absence of authentic standards, we utilized SCD1 inhibitor or FADS2 inhibitor to disturb cells and tracked compositional changes of these uncanonical FA C=C isomers. Additionally, the total FAs in the cell medium were profiled and later used to assess the influence of lipid uptake from the environment. To ensure the reliability and robustness of our experimental results, we performed analysis on six biological replicates, each accompanied by three technical replicates. The average coefficient of variation was less than 1% across three technical replicates and less than 8% across six biological replicates.

### Profiling of total FAs at the sum composition level

**Fig. 2:**
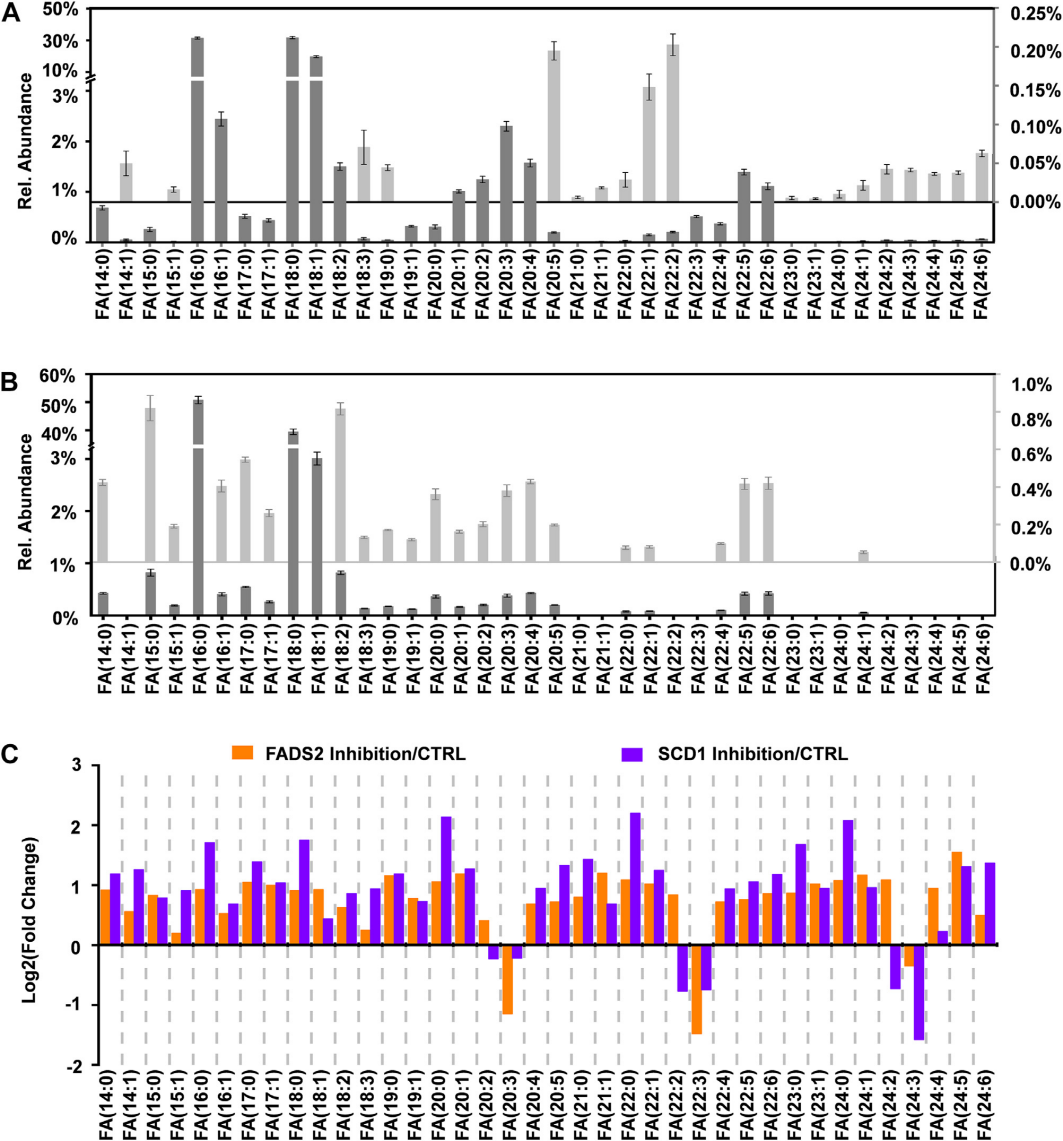
Relative abundances of total FA at the sum composition level in (A) RAW 264.7 cells and (B) the culture medium. C: fold changes of total FAs induced by inhibition of SCD1 or FADS2 relative to the control.

A total of 29 groups of unsaturated FA and 9 groups of saturated FA were identified at the sum composition level, covering long-chain (carbon number: 14–21) and very-long-chain FAs (carbon number >21) with up to 6 C=Cs. Our data (Fig. 2A) showed a comparable profile to the free FAs (a total of 31 FAs) detected in RAW 264.7 macrophages via GC/EI-MS (40). FA 16:0 (31.5 ± 0.7%), FA 18:1 (19.8 ± 0.7%), and FA 18:0 (31.8 ± 0.7%) were the most abundant FAs. MUFAs containing odd-carbon number chains, such as FA 15:1 (0.016 ± 0.003%), FA 17:1 (0.43 ± 0.03%), FA 19:1 (0.32 ± 0.02%), and FA 21:1 (0.018 ± 0.001%), were present at relative abundances 2-3 orders of magnitude lower than that of FA 18:0 (Fig. 2A). Furthermore, we conducted analysis of the total FAs in the culture medium, revealing that FA 16:0 (50.8 ± 1.3%) and FA 18:0 (39.5 ± 1.0%) as the most abundant FAs. Notably, FA 18:1 (3.0 ± 0.1%) represented the only other FA with a relative abundance exceeding 1%, while the remaining FAs all exhibited relative abundances below 1% (Fig. 2B). Inhibition of SCD1 and FADS2 enzymes resulted in a significant increase in most FAs at the sum composition level, which might result from more active uptake of fatty acids from the culture medium (Fig. 2C and supplemental Table S1). Only a subset of fatty acids, such as FA 20:3, 22:3, and 24:3, demonstrated a notable decrease (Fig. 2C).

The retention time (RT) values of AMPP-derivatized FAs were plotted as a function of carbon chain length (14−24) and the number of C=C (0−6) (supplemental Fig. S1). We clearly observe two distinct chromatographic peaks for FA 15:0, FA 17:0, and FA 19:0, respectively. Based on the RT trendline and the literature report (41), the later eluting peaks should correspond to the straight-chain fatty acids, while the earlier eluting peaks might represent their corresponding branched-chain fatty acid isomers. However, even with prolonged separation time (50 min), only a single chromatographic peak was detected for each odd-chain MUFA. Consequently, this investigation was unable to distinguish between straight MUFAs and branched-chain MUFAs.

### Profiling of odd-chain MUFAs

**Fig. 3:**
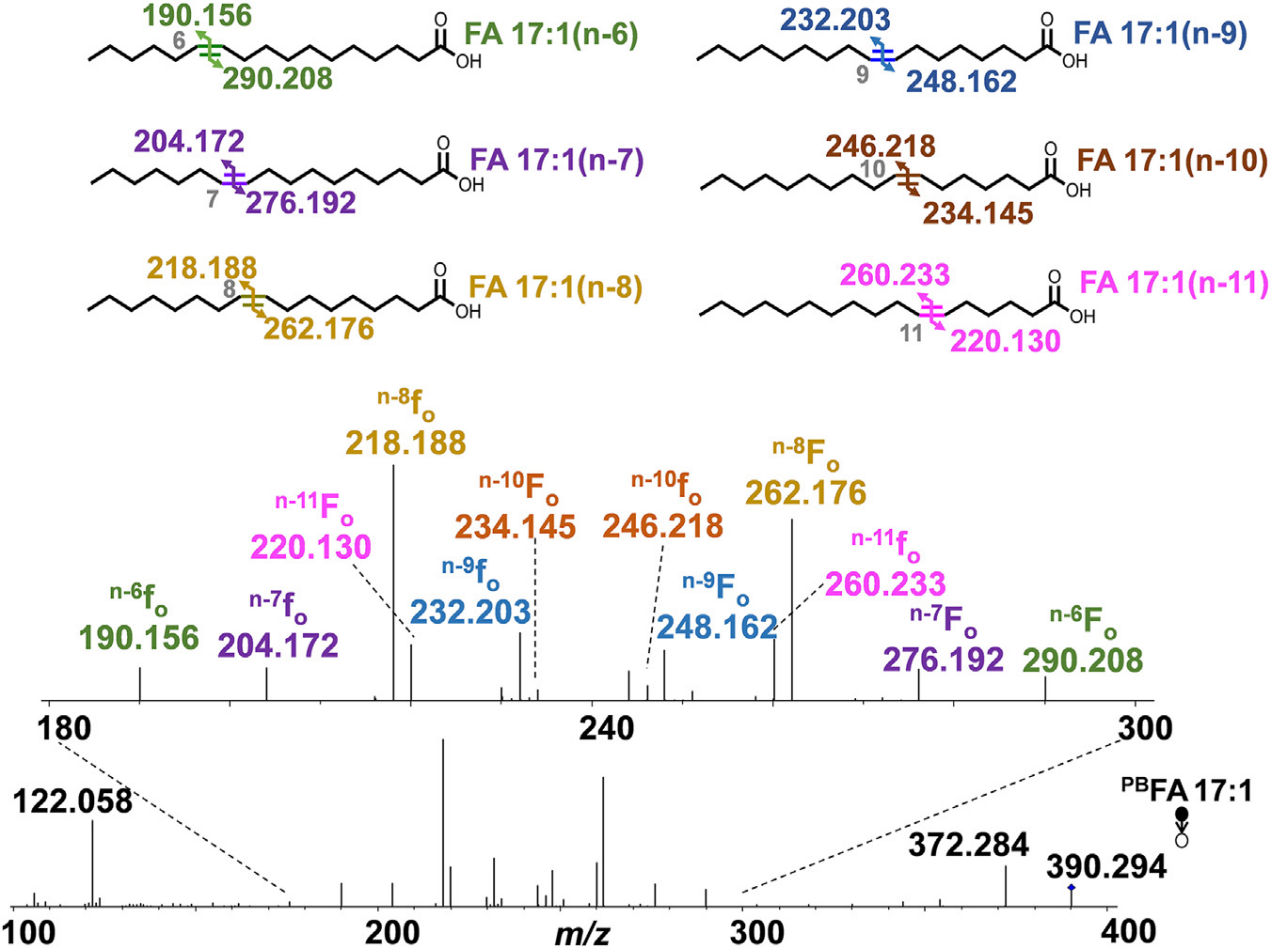
MS^2^ CID spectrum of 2-acpy modified FA 17:1 ([^PB^FA+H]^+^, m/z 390.3). Fragmentation maps of the six detected C=C location isomers of FA 17:1 (n-6, n-7, n-8, n-9, n-10, and n-11) are shown in the insets.

Since the odd-chain MUFAs and their C=C location isomers have been rarely characterized, we focused our attention to analyze the odd-chain MUFAs. Figure 3 shows the PB-MS/MS spectrum of FA 17:1. Based on detecting the C=C diagnostic ions (^n-x^f_o_ and ^n-x^F_o_, generic structures shown in Fig. 1), we confidently identified six C=C location isomers, including a predominant n-8 isomer (56 ± 3%), followed by the n-11 (15.3 ± 0.8%), n-9 (14.9 ± 1.8%), n-7 (5.7 ± 1.4%), n-6 (4.4 ± 0.2%), and n-10 (4.0 ± 0.6%) isomers (supplemental Tables S2 and S3). %Relative composition was calculated by normalizing the relative abundances of the C=C diagnostic ions of a certain isomer to the summed abundances of the diagnostic ions from all C=C location isomers.

**Fig. 4:**
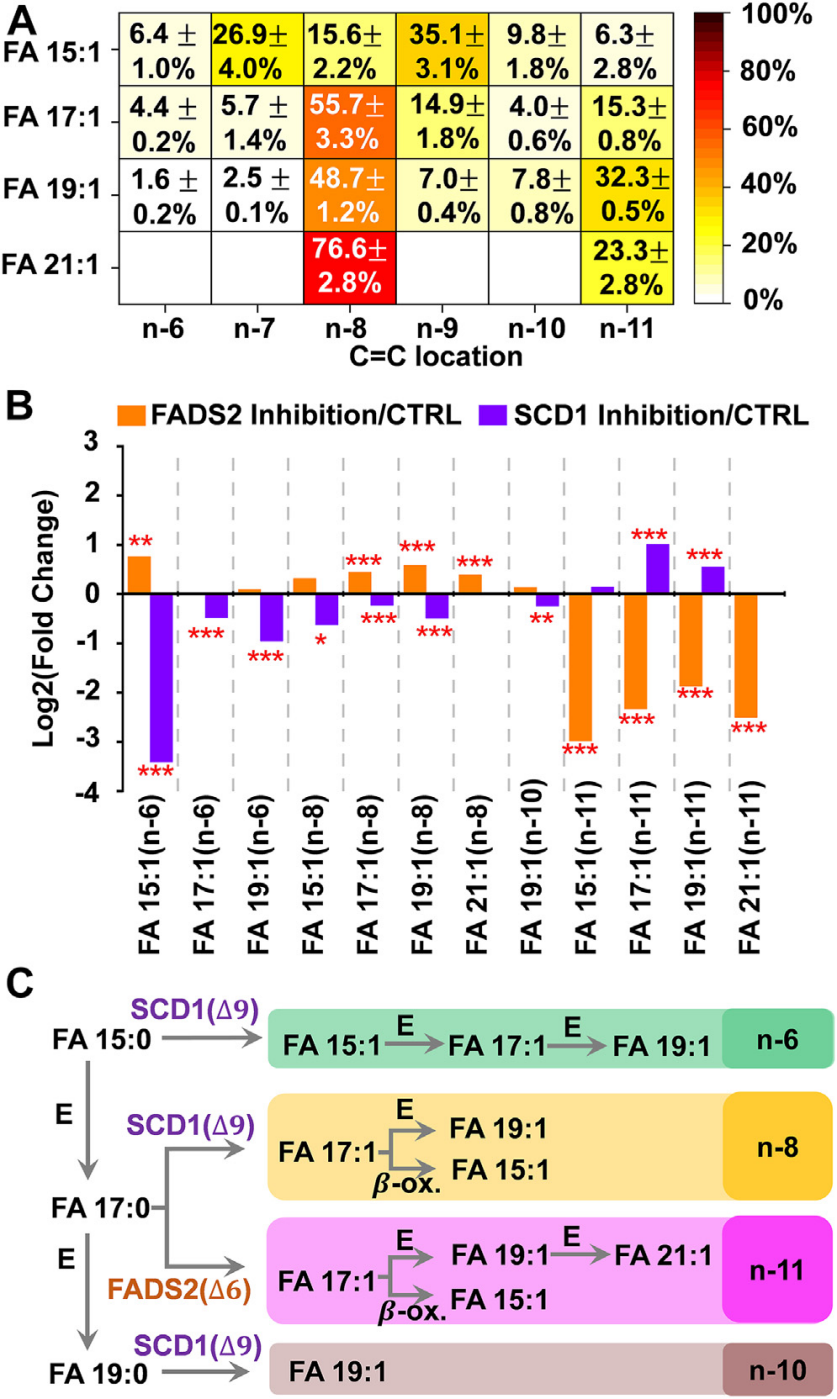
A: %relative compositions of C=C location isomers of FA 15:1, FA 17:1, and FA 19:1 in RAW 264.7 cells. B: Fold changes in the odd-chain MUFA levels induced by inhibition of the SCD1 or the FADS2 relative to the control. Differences between the two groups of samples were evaluated for statistical significance using the two-tailed student's *t* test (∗*P* < 0.05, ∗∗*P* < 0.01, ∗∗∗*P* < 0.001). C: Proposed biosynthetic pathways of the odd-chain MUFAs. E: elongation; β-ox.: β-oxidation.

Figure 4A summarizes the distribution of C=C location isomers of FA 15:1, FA 17:1, FA 19:1, and FA 21:1 in RAW 264.7 cells. Substantial FA 19:1 (n-8) was detected (48.7 ± 1.2%), which might be generated from elongation of FA 17:1(n-8) (56 ± 3%) (Fig. 4A and supplemental Fig. S2). Recently, the Brenna group demonstrated that FA 17:1(n-8) could be generated by SCD1 Δ9-desaturation of FA 17:0 (15). The β-oxidation product of FA 17:1(n-8), viz., FA15:1(n-8) (15.6 ± 2.2%), was also detected (Fig. 4A). To confirm that the n-8 isomers of the odd-chain MUFAs are indeed the desaturation products of SCD1, we treated RAW 264.7 cells with CAY10566, a small-molecule inhibitor of SCD1. Figure 4B shows that inhibition of SCD1 reduces the formation of the n-8 families of the odd-chain MUFAs, including FA 15:1(n-8), FA 17:1(n-8), and FA 19:1(n-8). For instance, the %relative composition of FA 17:1 (n-8) decreased from 56 ± 3% to 47.6 ± 1.0% (*P* = 1.6×10^−4^). These data corroborate that SCD1 is involved in de novo synthesis of these n-8 isomers. However, the %relative composition of FA 21:1 (n-8) showed an increase after SCD1 inhibition (from 77 ± 3% to 100.0 ± 0%, *P* = 1.8 ×10^−9^). Because FA 21:1(n-8) is the single component found in the culture medium for FA 21:1 (supplemental Fig. S3 and supplemental Tables S3 and S4), we thus hypothesize that FA 21:1(n-8) in the cells is mainly contributed by medium uptake instead of de novo synthesis. The associate data of Fig. 4B are provided in supplemental Table S3.

We also observed a significant decrease in %relative composition of the n-6 isomers of FA 15:1, FA 17:1, and FA 19:1, along with FA 19:1(n-10), after the inhibition of SCD1 (Fig. 4B). It is likely that SCD1 mediates FA 15:0 → FA 15:1(n-6) via Δ9-desaturation and promotes FA 19:0 → FA 19:1(n-10) (Fig. 4C and supplemental Fig. S2). The small contribution of FA 17:1(n-6) (4.4 ± 0.2%) might result from elongation of FA 15:1 (n-6) (6.4 ± 1.0%). Subsequent elongation of FA 17:1 (n-6) gives rise to FA 19:1 (n-6) (1.6 ± 0.2%).

After FADS2 inhibition, the relative compositions of the n-11 isomers of the odd-chain MUFAs decreased significantly (Fig. 4B), such as FA 15:1(n-11), FA 17:1(n-11), FA 19:1(n-11), and FA 21:1(n-11). Interestingly, FADS2 inhibition also consolidated SCD1 activity, rendering a prevalent increase in most n-6 and n-8 odd-chain MUFAs isomers, such as FA 15:1(n-6), FA 17:1(n-8), and FA 19:1(n-8).

As discussed earlier, FA 17:1 only accounted for 0.43 ± 0.03% of the total FAs in RAW 264.7 cells at the sum composition level, while enzymatic inhibition showed small disturbances to its relative abundance, eg, 0.33 ± 0.03% in the SCD1 inhibition group and 0.47 ± 0.04% in the FADS2 inhibition group (Fig. 2A). The analysis at the C=C location level showed no significant difference in the relative compositions of FA 17:1 (n-7), 17:1 (n-9), and 17:1 (n-10) following either SCD1 inhibition or FADS2 inhibition. On the other hand, FA 17:1 accounted for 0.26 ± 0.02% of the total FAs in the culture medium, while the three FA 17:1 isomers were detected with %relative compositions at 3.3 ± 0.4% (n-7), 18.1 ± 1.2% (n-9), and 3.5 ± 0.1% (n-10) (supplemental Fig. S3 and supplemental Table S4), respectively. These values are comparable to those detected in RAW264.7 cells, e.g., FA 17:1 (n-7) (5.7 ± 1.4%), 17:1 (n-9) (14.9 ± 1.8%), and 17:1 (n-10) (4.0 ± 0.6%). We hypothesize that the FA 17:1 isomers are mainly derived from the culture medium; however, the extent of contribution from the culture medium cannot be quantified without performing isotopic tracing experiments.

### Profiling of even-chain MUFAs

**Fig. 5:**
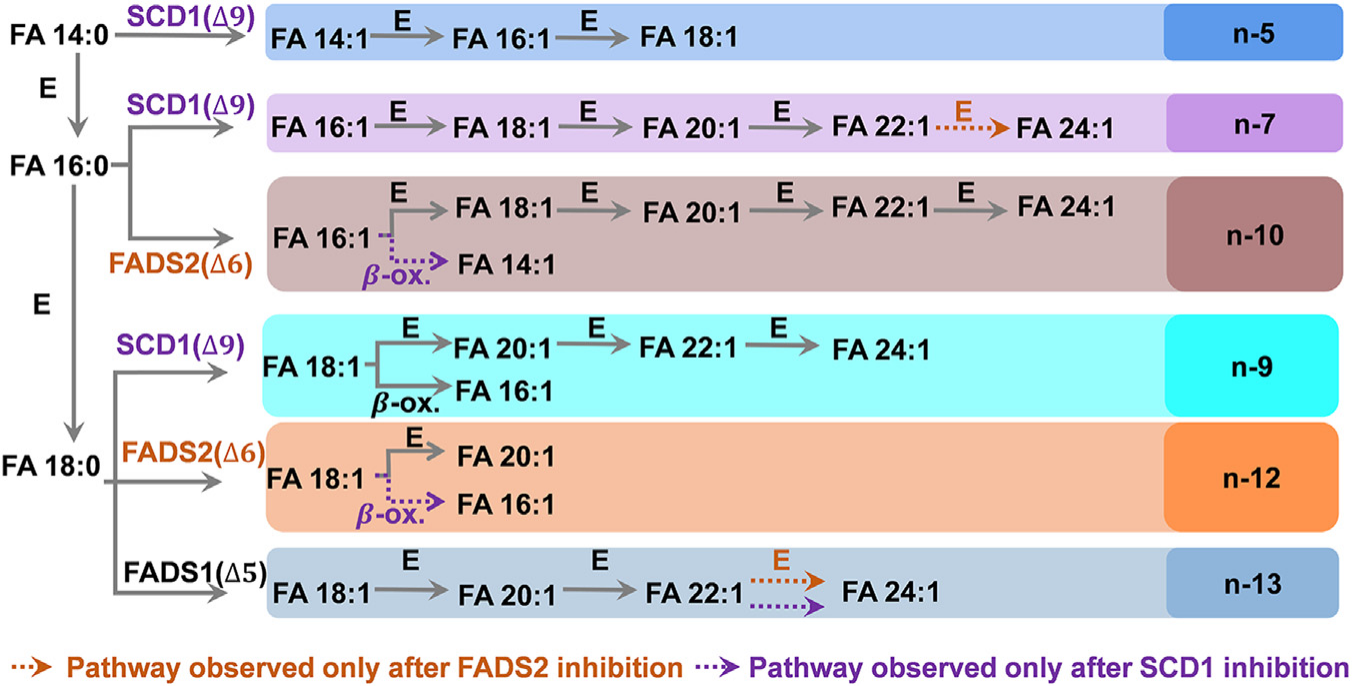
Proposed biosynthetic pathways of even-chain MUFAs observed in RAW cells. E: elongation; β-ox.:β-oxidation.

Consistent with a previous report by Blanksby and co-workers (13), Δ9 desaturation of FA 14:0, FA 16:0, and FA 18:0 by SCD1 generates n-5, n-7, and n-9 families of the even-chain MUFAs. Notably, we observed successive elongations of FA 16:1(n-7) to FA 18:1 (n-7) and further to FA 20:1(n-7) and FA 22:1 (n-7). A similar chain elongation was observed in FA 18:1(n-9), showing the existence of the n-9 isomers of FA 20:1, FA 22:1, and FA 24:1 (supplemental Fig. S4A). Inhibition of SCD1 prevented the formation of n-5, n-7, and n-9 families of even-chain MUFAs from undergoing SCD1 desaturation to FA 14:0, FA 16:0, and FA 18:0 and thus consolidated desaturation activity through FADS2 and FADS1. This, in turn, led to an increase in the n-10, n-12, and n-13 isomers in the even-chain MUFAs families (supplemental Fig. S4B). For instance, inhibition of SCD1 led to a pronounced elevation in the relative composition of FA 22:1 (n-13), rising from 53 ± 4% to 81.2 ± 1.2%. FA 16:1 (n-12) was not detected in the control group (supplemental Fig. S4A); however, its relative composition was found at 3.6 ± 0.3% in the SCD1 inhibition group (supplemental Table S3). Upregulation of the n-10, n-12, and n-13 isomers can be ascribed to the Δ6-desaturation of FA 16:0 and FA 18:0 by FADS2 and Δ5-desaturation of FA 18:0 by FADS1 due to reduced competition by SCD1 for the same substrates. Several types of cancer cells were found to synthesize FA 16:1(n-10) via FADS2 catalysis (42) when the activity of SCD1 was compromised, in line with our observations here. FADS2 desaturation can yield either n-10 or n-12 FAs depending on the substrate. This is supported by detecting decreased n-10 and n-12 isomers in the even-chain MUFAs after FADS2 inhibition (*P* <0.001, N = 6) (supplemental Fig. S4B). The proposed pathways of even-chain MUFAs observed in RAW 264.7 cells are summarized in Fig. 5.

### Profiling of PUFAs

**Fig. 6:**
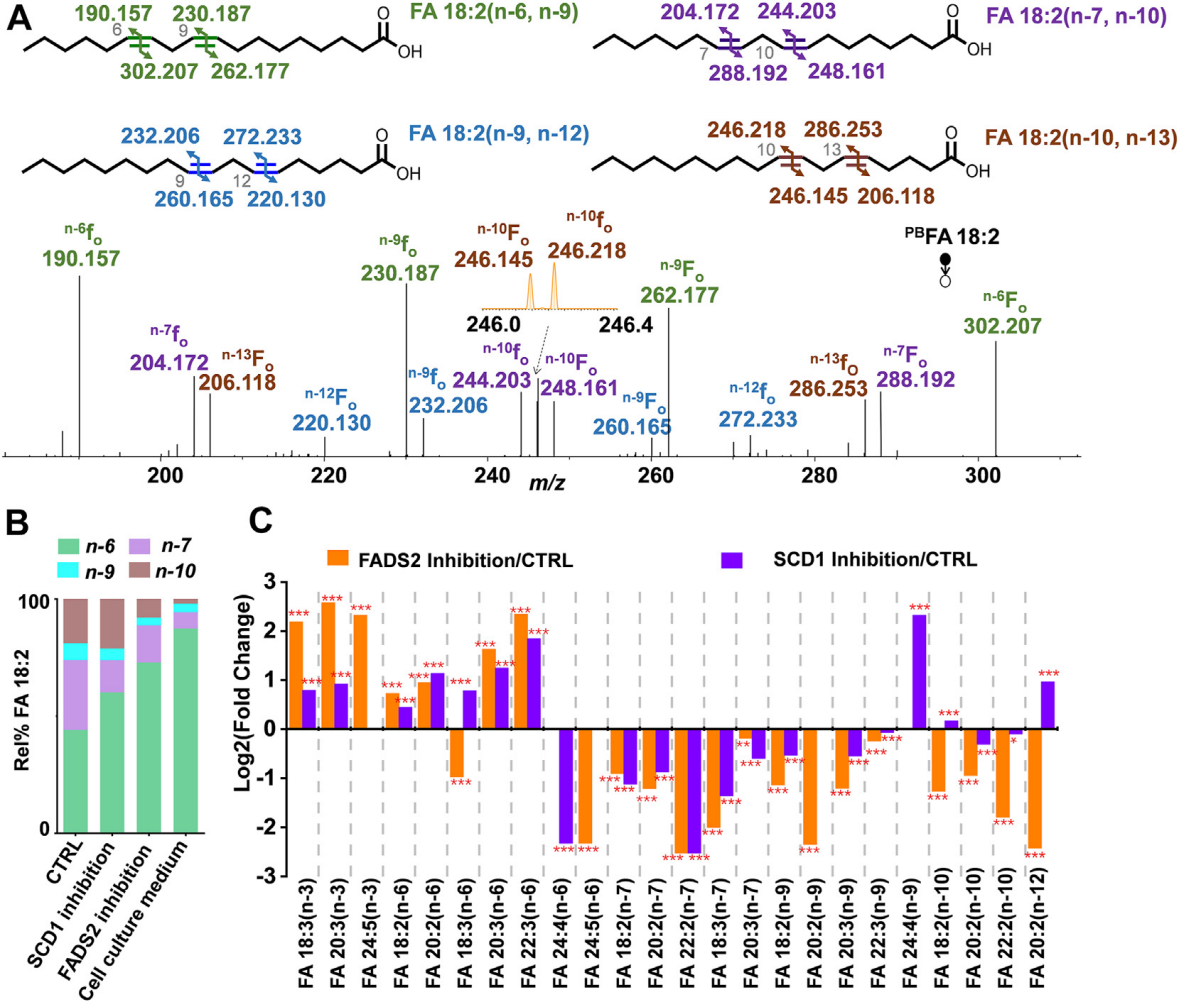
A: PB-MS^2^ CID spectrum of 2-acpy modified FA 18:2 in RAW 264.7 cells. Fragmentation maps of FA 18:2(n-6, n-9), FA 18:2(n-7, n-10), FA 18:2(n-9, n-12) and FA 18:2(n-10, n-13) are shown in the insets. B: Comparison of %relative compositions of FA 18:2 C=C location isomers between RAW 264.7 cells with and without inhibition of the SCD1 enzyme or the FADS2 enzyme and cell culture medium. C: Fold changes in PUFA levels induced by inhibition of the SCD1 enzyme or the FADS2 enzyme relative to the control measured in RAW 264.7 cells. The PUFAs depicted herein are all methylene-interrupted. Differences between the two groups of samples were evaluated for statistical significance using the two-tailed student's *t* test (∗*P* < 0.05, ∗∗*P* < 0.01, ∗∗∗*P* < 0.001).

Besides MUFA diversification, de novo synthesis of PUFAs in RAW 264.7 cells proved to be significantly more intricate than originally anticipated. Figure 6A shows the PB-MS^2^ CID spectrum of FA 18:2. Based on detecting the C=C diagnostic ions (^n-x^f_o_ and ^n-x^F_o_), FA 18:2 was identified as a mixture of FA 18:2 (n-6, n-9) (44.2 ± 0.8%), FA 18:2 (n-7, n-10) (29.8 ± 0.5%), FA 18:2(n-9, n-12) (7.2 ± 0.6%), and FA 18:2(n-10, n-13) (18.9 ± 0.3%). The presence of FA 18:2 (n-7, n-10) indicates that FADS2 catalyzes Δ8-desaturation of FA 18:1(n-7) to form FA 18:2(n-7, n-10). Further chain elongation and Δ5-desaturation of FA 18:2(n-7, n-10) contribute to the n-7 PUFAs identified in FA 20:2, FA 22:2, FA 18:3, and FA 20:3 (supplemental Figs. S5 and S6). FA 18:3 was typically observed as a mixture of FA 18:3(n-3, n-6, n-9) and FA 18:3(n-6, n-9, n-12) in mammalian lipidome (31, 43, 44, 45). However, in RAW 264.7 cells the most abundant isomer was FA 18:3(n-7, n-10, n-13), accounting for 54.2 ± 0.8%, followed by the n-6 (30.2 ± 0.5%) and n-3 (15.6 ± 0.6%) isomers (supplemental Fig. S5). Considering the involvement of FADS2 and SCD1 in the formation of the n-7 families PUFA, an inhibition of FADS2 or SCD1 should both decrease the level of n-7 families of PUFAs. For instance, %relative composition of FA 18:2 (n-7, n-10) decreased from 29.8 ± 0.5% in control to 13.8 ± 1.2% after SCD1 inhibition (*P* = 3.7×10^−11^) (Fig. 6B). Similarly, %relative composition of FA 18:2(n-7, n-10) decreased from 29.8 ± 0.5% in control to 16.0 ± 0.8% in FADS2 inhibition samples (*P* = 9.7×10^−12^) (Fig. 6B). A similar phenomenon was also observed for the other n-7 families of PUFAs, including FA 20:2 (n-7, n-10), FA 22:2 (n-7, n-10), FA 18:3 (n-7, n-10, n-13) and FA 20:3(n-7, n-10, n-13) (Fig. 6C)

Besides the n-7 families of PUFAs, the n-9 families of PUFA were also identified. The presence of FA 18:2 (n-9, n-12) indicates that FADS2 catalyzes Δ6-desaturation of FA 18:1(n-9) to form FA 18:2(n-9, n-12). Furthermore, the n-9 isomer was found to be the most dominant form in FA 20:3 (73.9 ± 0.5%), which is an indicator of essential fatty acid deficiency (46). Previous studies reported that SCD1, FADS2, and FADS1 were involved in the synthesis of FA 20:3 (n-9, n-12 ,n-15) from FA 18:0, and two pathways were involved: i) FA 18:0 $\overset{SCD1,\Delta 9}{\to }$ FA 18:1(n-9) $\overset{FADS2,\Delta 6}{\to }$ FA 18:2(n-9, n-12) $\overset{E}{\to }$ FA 20:2(n-9, n-12) $\overset{FADS1,\Delta 5}{\to }$ FA 20:3(n-9, n-12, n-15) and ii) FA 18:0 $\overset{SCD1,\Delta 9}{\to }$ FA 18:1(n-9) $\overset{E}{\to }$ FA 20:1(n-9) $\overset{FADS2,\Delta 8}{\to }$ FA 20:2(n-9, n-12) $\overset{FADS1,\Delta 5}{\to }$ FA 20:3(n-9, n-12, n-15) (46). An inhibition of FADS2 or SCD1 significantly decreased the abundance of the n-9 isomers of PUFAs. For instance, %relative composition of FA 18:2(n-9, n-12) decreased from 7.2 ± 0.5% to 5.0 ± 0.7% after SCD1 inhibition (*P* =1.5×10^−4^) and 3.3 ± 1.1% after FADS2 inhibition (*P* =1.9×10^−5^) (Fig. 6B). A similar trend was observed among other n-9 families of PUFAs, including FA 20:2(n-9, n-12), FA 18:3(n-9, n-12, n-15), and FA 20:3(n-9, n-12, n-15; Fig. 6C).

Likewise, the presence of FA 18:2(n-10, n-13) indicates the possibility for Δ5-desaturation of FA 18:1(n-10) by FADS1, which highlights a point of divergence in enzymatic activity surrounding the FA 18:1(n-10). The subsequent elongation of FA 18:2(n-10, n-13) results in the n-10 PUFAs, including FA 20:2(n-10, n-13) and FA 22:2(n-10, n-13). A similar enzyme branching point can be observed for FA 20:1(n-12), whereby FADS1 mediates Δ5-desaturation towards FA 20:1(n-12) → FA 20:2(n-12, n-15). Thus inhibition of the FADS2 leads to a decrease in the abundance of n-10 and n-12 isomers of PUFAs, including FA 18:2(n-10, n-13), FA 20:2(n-10, n-13), FA 22:2(n-10, n-13) and FA 20:2(n-12, n-15) (Fig. 6C).

**Fig. 7:**
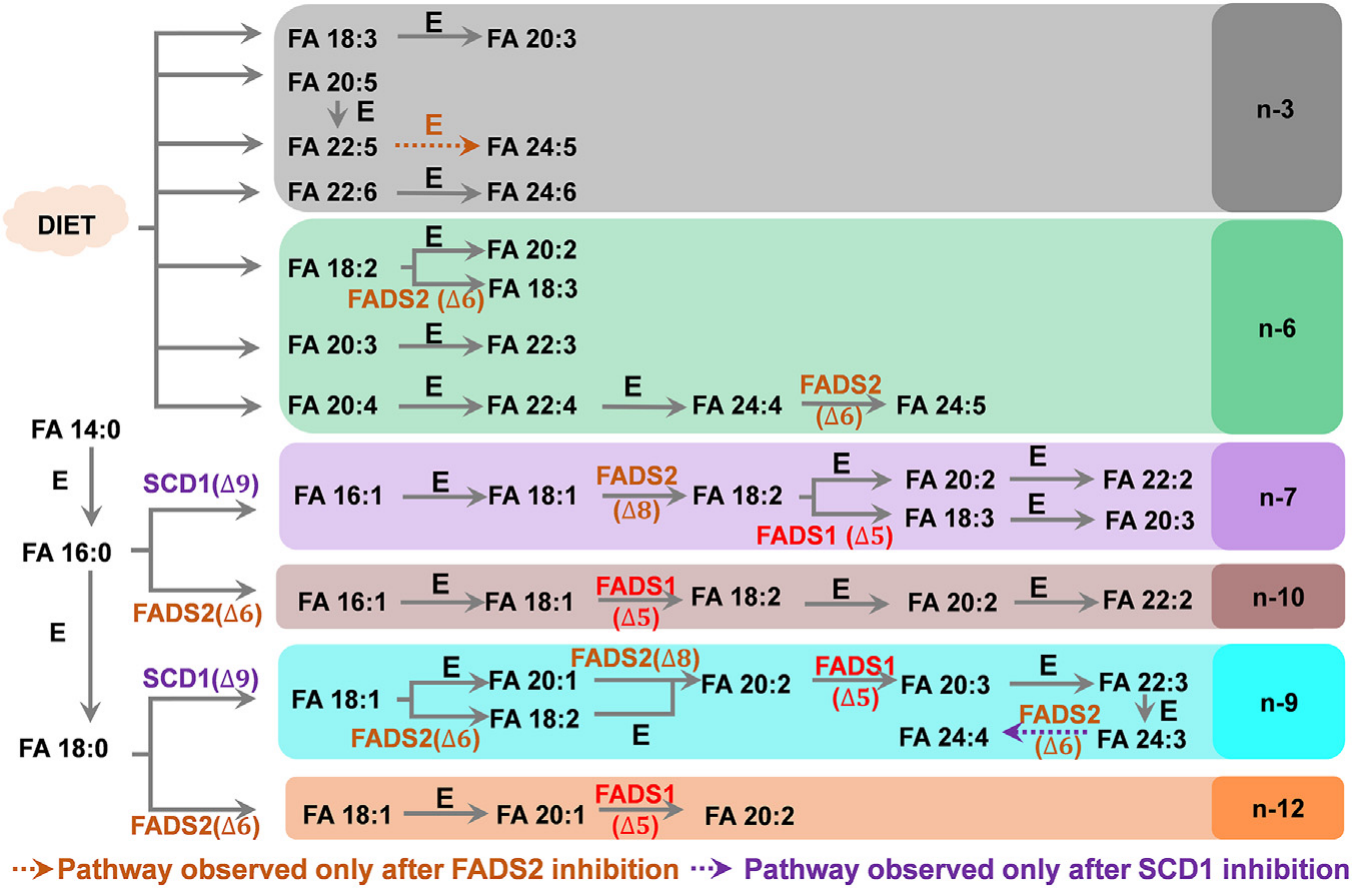
Biosynthetic pathways of PUFAs proposed for RAW 264.7 cells. E: elongation; β-ox.:β-oxidation.

RAW 264.7 cells tend to uptake n-3 and n-6 PUFAs from the culture medium when the de novo synthesis pathways for n-7, n-9, n-10, and n-12 PUFAs are blocked. In the culture medium, the n-3 isomers dominated in FA 18:3 (95.6 ± 0.2%), FA 20:5 (100 ± 0%), FA 22:5 (100 ± 0%) and FA 22:6 (100 ± 0%), and the n-6 isomers dominated in FA 18:2 (87.5 ± 0.8%), FA 20:3 (75.3 ± 0.8%), FA 20:4 (92.9 ± 0.1%), and 22:4 (64 ± 4%) (supplemental Fig. S3 and supplemental Tables S3 and S4). The relative compositions of FA 18:3(n-3, n-6, n-9), FA 20:3(n-3, n-6, n-9), FA 18:2(n-6, n-9), FA 20:2(n-6, n-9), FA 20:3(n-6, n-9, n-12), and FA 22:3(n-6, n-9, n-12) in RAW 264.7 cells all significantly increased after inhibition of FADS2 or SCD1 (*P* <0.001, N = 6), except for FA 18:3(n-6, n-9, n-12) and FA 24:5(n-6, n-9, n-12, n-15, n-18) (Fig. 6C). Interestingly, the n-3 isomers of FA 22:5 and FA 22:6 were consistently observed as the only component (100%) of corresponding PUFAs in the culture medium, control, SCD1-inhibition, and FADS2-inhibition groups (supplemental Fig. S6). We thus hypothesize that culture medium is the only source for these two PUFAs. On the other hand, the n-3 isomer of FA 24:6 was not detected in the culture medium; yet it was the only component (100%) detected in the control, SCD1-inhibition, and FADS2-inhibition groups (supplemental Fig. S6). This phenomenon indicates that the n-3 isomer of FA 24:6 may be the elongation product of FA 22:6 (n-3) (Fig. 7).

FADS2 can convert FA 18:2(n-6, n-9) to FA 18:3(n-6, n-9, n-12) and convert FA 24:4 (n-6, n-9, n-12, n-15) to FA 24:5 (n-6, n-9, n-12, n-15, n-18) by catalyzing the desaturation at the Δ6 position (47). Thus, an inhibition of FADS2 decreased the abundances of FA 18:3(n-6, n-9, n-12) and FA 24:5(n-6, n-9, n-12, n-15, n-18) (Fig. 6c). In FADS2 inhibition samples, the %relative composition of FA 18:3(n-6, n-9, n-12) was 15.4 ± 0.8%, which was notably lower than the 30.2 ± 0.5% observed in the control (*P* = 2.3×10^−12^). In the control group, FA 24:4 only contained the n-6 isomer (100%), likely originating from sequential elongation of the n-6 isomer of FA 20:4 (97.2 ± 1.4%) to FA 22:4 (85 ± 3%). The n-6 isomer of FA 24:5 was observed as the only component (100%) in the control and SCD1-inhibition groups but absent in the FADS2-inhibition group and culture medium (supplemental Fig. S6). These data provide strong support to the pathway depicted in Fig. 7 that FA 24:5 (n-6, n-9, n-12, n-15, n-18) is formed from FADS2 desaturation of FA 24:4 (n-6, n-9, n-12, n-15).

Note that the Δ6-desaturase activity of FADS2 was greatly increased by SCD1 inhibition in the conversion of FA 24:3 (n-9, n-12, n-15) to FA 24:4 (n-9, n-12, n-15, n-18). %Relative composition of FA 24:4 (n-9, n-12, n-15, n-18) increased from 0% in control to 100% in after SCD1 inhibition. These results also confirm that SCD1 inhibition consolidates FADS2 activity toward PUFA. The biosynthetic pathways of PUFA observed in RAW cells are proposed in Fig. 7.

**Fig. 8:**
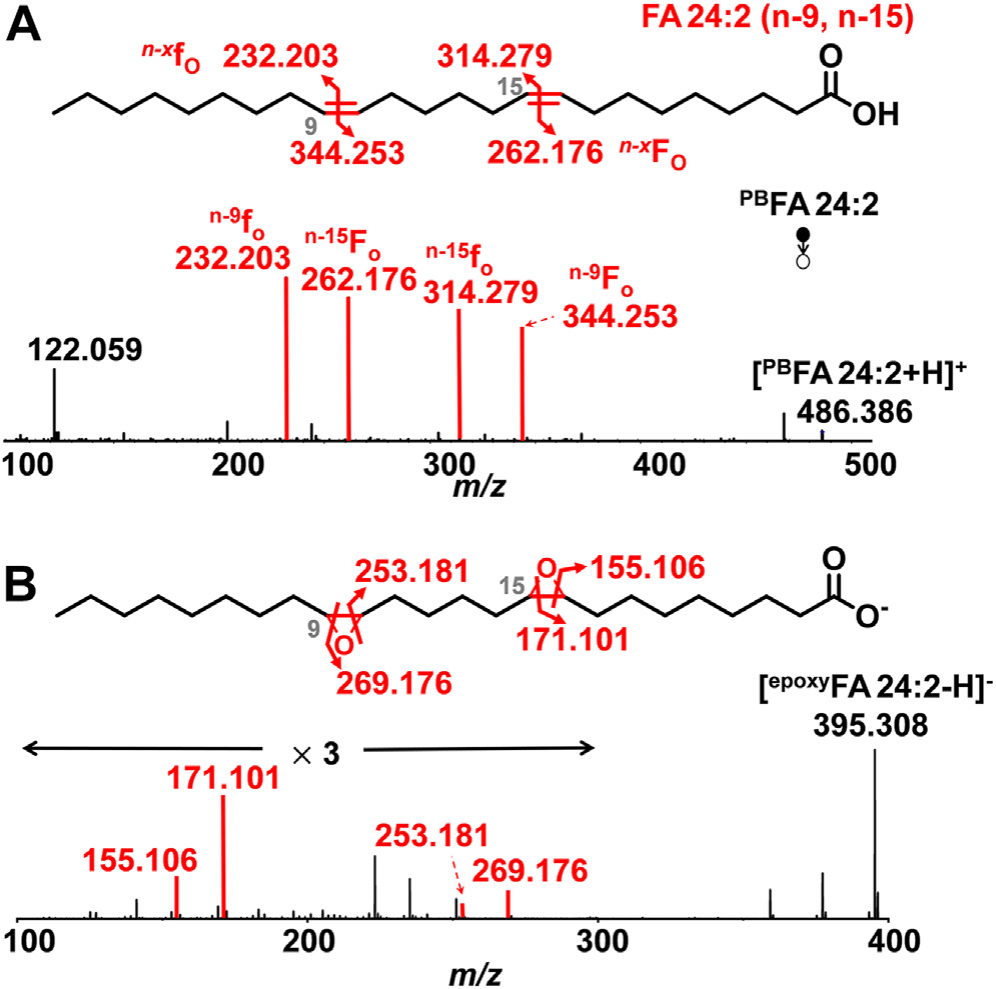
MS^2^ CID spectra of (A) 2-acpy modified FA 24:2 and (B) epoxidation product of FA 24:2 in RAW 264.7 cells. Fragmentation maps of FA 24:2(n-9, n-15) are shown in the insets.

Notably, we also identified two non-methylene-interrupted FAs in RAW 264.7 cells, viz., FA 22:2(n-9, n-15) (72.2 ± 0.4%) and FA 24:2 (n-9, n-15) (100%) (Fig. 8A and supplemental TableS1). The structure of FA 24:2(n-9, n-15) was further validated by m-CPBA epoxidation and RPLC-MS^2^ CID (Fig. 8B). Similarly, Menzel *et al.* detected the n-9, n-15 C=C locations in FA 22:2 and FA 24:2 through the OzID technique in MCF7 breast cancer cells (48). Non-methylene-interrupted FAs have been found in mollusks, with the most commonly occurring FA 20:2 (n-9, n-15) or FA 22:2 (n-9, n-15), constituting up to 20% of total FAs (49). De novo lipogenesis of the non-methylene-interrupted PUFAs and their precise biological functions are not well-known, although it has been suggested that they may compete with n-3 PUFAs for incorporation into polar lipids (49).

## Discussion

The exploration of FA C=C isomers within RAW 264.7 cell lipidome in combination with enzyme inhibition reveals that cellular fatty acid desaturation is far more complex and dynamic than previously considered. The biosynthesis pathways of the odd-chain MUFAs, even-chain MUFAs, and PUFAs are proposed based on the experimental observations. This expanded network of FA desaturation provides plasticity to generate certain FA families arising from extreme metabolic stress. For instance, the FADS2 plasticity allows for multiple substrate acceptances (e.g., FADS2 Δ 6-desaturation of FA 17:0 to yield FA 17:1 n-11) or an alternative site of desaturation. Similarly, under metabolic stress, SCD1 also displays plasticity toward substrate preference to allow for the formation of n-6, n-8, and n-10 of the odd-chain MUFAs and n-5, n-7, and n-9 of the even-chain MUFA families. The de novo synthesis of n-7, n-9, n-10, and n-12 PUFAs is worth attention, given their structural similarity to the dietary-derived and biologically active FAs, such as arachidonic acid. They may disrupt homeostatic lipid signaling or fulfill signaling roles in the absence of dietary FA uptake or upon other chemical environment changes. Notably, when de novo lipogenesis of n-7, n-9, n-10, and n-12 of PUFAs is hampered, cells can increase the uptake of dietary FAs (e.g., n-3 and n-6 of PUFAs) to meet metabolic demands. These are consistent with a recent report by the Blanksby group in which hundreds of fatty acid structures were discovered in MCF7 and LNCAP cancer cell lines via OzID (48). In conclusion, this study has revealed various new fatty acids in RAW 264.7 cells with unusual site(s) of unsaturation that are not described by canonical pathways. Although further work is required to specify the biological impact of the unusual lipid unsaturation, the experimental workflows and findings presented in the work can serve as a roadmap toward future discovery.

## Data availability

All data concerned with this study are presented within this manuscript.

## Supplemental data

This article contains supplemental data.

## Conflict of interest

The authors declare no conflicts of interest.

## References

[1] Harayama T., Riezman H. (2018). Understanding the diversity of membrane lipid composition. Nat. Rev. Mol. Cell Biol..

[2] Nakamura M.T., Yudell B.E., Loor J.J. (2014). Regulation of energy metabolism by long-chain fatty acids. Prog. Lipid Res..

[3] Marakalala M.J., Raju R.M., Sharma K., Zhang Y.J., Eugenin E.A., Prideaux B. (2016). Inflammatory signaling in human tuberculosis granulomas is spatially organized. Nat. Med..

[4] Ballweg S., Ernst R. (2017). Control of membrane fluidity: the OLE pathway in focus. Biol. Chem..

[5] Richard D., Kefi K., Barbe U., Bausero P., Visioli F. (2008). Polyunsaturated fatty acids as antioxidants. Pharmacol. Res..

[6] Ravaut G., Légiot A., Bergeron K.-F., Mounier C. (2020). Monounsaturated fatty acids in obesity-related inflammation. Int. J. Mol. Sci..

[7] Strittmatter P., Spatz L., Corcoran D., Rogers M.J., Setlow B., Redline R. (1974). Purification and properties of rat liver microsomal stearyl coenzyme A desaturase. Proc. Natl. Acad. Sci. U. S. A..

[8] Paton C.M., Ntambi J.M. (2009). Biochemical and physiological function of stearoyl-CoA desaturase. Am. J. Physiol. Endocrinol. Metab..

[9] Nakamura M.T., Nara T.Y. (2004). Structure, function, and dietary regulation of (delta) 6,(delta) 5, and (delta) 9 desaturases. Annu. Rev. Nutr..

[10] Beloribi-Djefaflia S., Vasseur S., Guillaumond F. (2016). Lipid metabolic reprogramming in cancer cells. Oncogenesis.

[11] Vriens K., Christen S., Parik S., Broekaert D., Yoshinaga K., Talebi A. (2019). Evidence for an alternative fatty acid desaturation pathway increasing cancer plasticity. Nature.

[12] Ottaviani M., Camera E., Picardo M. (2010). Lipid mediators in acne. Med. Inflamm..

[13] Young R.S.E., Bowman A.P., Williams E.D., Tousignant K.D., Bidgood C.L., Narreddula V.R. (2021). Apocryphal FADS2 activity promotes fatty acid diversification in cancer. Cell Rep..

[14] Brenna J.T., Kothapalli K.S.D. (2022). New understandings of the pathway of long-chain polyunsaturated fatty acid biosynthesis. Curr. Opin. Clin. Nutr. Metab. Care.

[15] Wang Z., Park H.G., Wang D.H., Kitano R., Kothapalli K.S.D., Brenna J.T. (2020). Fatty acid desaturase 2 (FADS2) but not FADS1 desaturates branched chain and odd chain saturated fatty acids. Biochim. Biophys. Acta.

[16] Quehenberger O., Armando A.M., Dennis E.A. (2011). High sensitivity quantitative lipidomics analysis of fatty acids in biological samples by gas chromatography–mass spectrometry. Biochim. Biophys. Acta.

[17] Ecker J., Scherer M., Schmitz G., Liebisch G. (2012). A rapid GC–MS method for quantification of positional and geometric isomers of fatty acid methyl esters. J. Chromatogr. B.

[18] Chiu H.-H., Kuo C.-H. (2020). Gas chromatography-mass spectrometry-based analytical strategies for fatty acid analysis in biological samples. J. Food Drug Anal..

[19] Wang Z., Wang D.H., Park H.G., Tobias H.J., Kothapalli K.S.D., Brenna J.T. (2019). Structural identification of monounsaturated branched chain fatty acid methyl esters by combination of electron ionization and covalent adduct chemical ionization tandem mass spectrometry. Anal. Chem..

[20] Poad B.L., Marshall D.L., Harazim E., Gupta R., Narreddula V.R., Young R.S. (2019). Combining charge-switch derivatization with ozone-induced dissociation for fatty acid analysis. J. Am. Soc. Mass. Spectrom..

[21] Fang M., Rustam Y., Palmieri M., Sieber O.M., Reid G.E. (2020). Evaluation of ultraviolet photodissociation tandem mass spectrometry for the structural assignment of unsaturated fatty acid double bond positional isomers. Anal. Bioanal. Chem..

[22] Narreddula V.R., McKinnon B.I., Marlton S.J., Marshall D.L., Boase N.R., Poad B.L. (2021). Next-generation derivatization reagents optimized for enhanced product ion formation in photodissociation-mass spectrometry of fatty acids. Analyst.

[23] Zhao Y., Zhao H., Zhao X., Jia J., Ma Q., Zhang S. (2017). Identification and quantitation of C=C location isomers of unsaturated fatty acids by epoxidation reaction and tandem mass spectrometry. Anal. Chem..

[24] Kuo T.-H., Chung H.-H., Chang H.-Y., Lin C.-W., Wang M.-Y., Shen T.-L. (2019). Deep lipidomics and molecular imaging of unsaturated lipid isomers: a universal strategy initiated by mCPBA epoxidation. Anal. Chem..

[25] Yang T., Tang S., Kuo S.-T., Freitas D., Edwards M., Wang H. (2022). Lipid mass tags via aziridination for probing unsaturated lipid isomers and accurate relative quantification. Angew. Chem. Int. Ed. Engl..

[26] Zhang B., Wang Y., Zhou B.-W., Cheng J., Xu Q., Zhang L. (2022). Chloramine-T-Enabled mass spectrometric analysis of C=C isomers of unsaturated fatty acids and phosphatidylcholines in human thyroids. Anal. Chem..

[27] Ma X., Xia Y. (2014). Pinpointing double bonds in lipids by Paterno-Buchi reactions and mass spectrometry. Angew. Chem. Int. Ed. Engl..

[28] Ma X., Chong L., Tian R., Shi R., Hu T.Y., Ouyang Z. (2016). Identification and quantitation of lipid C=C location isomers: a shotgun lipidomics approach enabled by photochemical reaction. Proc. Natl. Acad. Sci. U.S.A..

[29] Xia T., Ren H., Zhang W., Xia Y. (2020). Lipidome-wide characterization of phosphatidylinositols and phosphatidylglycerols on CC location level. Anal. Chim. Acta.

[30] Xia T., Yuan M., Xu Y., Zhou F., Yu K., Xia Y. (2021). Deep structural annotation of glycerolipids by the charge-tagging paterno–büchi reaction and supercritical fluid chromatography–ion mobility mass spectrometry. Anal. Chem..

[31] Zhao J., Fang M., Xia Y. (2021). A liquid chromatography-mass spectrometry workflow for in-depth quantitation of fatty acid double bond location isomers. J. Lipid Res..

[32] Liebisch G., Fahy E., Aoki J., Dennis E.A., Durand T., Ejsing C.S. (2020). Update on LIPID MAPS classification, nomenclature, and shorthand notation for MS-derived lipid structures. J. Lipid Res..

[33] Matyash V., Liebisch G., Kurzchalia T.V., Shevchenko A., Schwudke D. (2008). Lipid extraction by methyl-tert-butyl ether for high-throughput lipidomics. J. Lipid Res..

[34] Zhao J., Xie X., Lin Q., Ma X., Su P., Xia Y. (2020). Next-Generation paternò–büchi reagents for lipid analysis by mass spectrometry. Anal. Chem..

[35] Xia T., Zhou F., Zhang D., Jin X., Shi H., Yin H., Gong Y. (2023). Deep-profiling of phospholipidome via rapid orthogonal separations and isomer-resolved mass spectrometry. Nat. Commun..

[36] Remmerie A., Scott C.L. (2018). Macrophages and lipid metabolism. Cell Immunol.

[37] Dennis E.A., Deems R.A., Harkewicz R., Quehenberger O., Brown H.A., Milne S.B. (2010). A mouse macrophage lipidome. J. Biol. Chem..

[38] Andreyev A.Y., Fahy E., Guan Z., Kelly S., Li X., McDonald J.G. (2010). Subcellular organelle lipidomics in TLR-4-activated macrophages 1 [S]. J. Lipid Res..

[39] Zhang D., Lin Q., Xia T., Zhao J., Zhang W., Ouyang Z. (2022). LipidOA: a machine-learning and prior-knowledge-based tool for structural annotation of glycerophospholipids. Anal. Chem..

[40] Quehenberger O., Armando A., Dumlao D., Stephens D.L., Dennis E.A. (2008). Lipidomics analysis of essential fatty acids in macrophages. Prostaglandins Leukot. Essent. Fatty Acids.

[41] Christinat N., Morin-Rivron D., Masoodi M. (2016). High-Throughput quantitative lipidomics analysis of nonesterified fatty acids in human plasma. J. Proteome Res..

[42] Snaebjornsson M.T., Schulze A. (2019). Tumours use a metabolic twist to make lipids. Nature.

[43] Wang M., Han R.H., Han X. (2013). Fatty acidomics: global analysis of lipid species containing a carboxyl group with a charge-remote fragmentation-assisted approach. Anal. Chem..

[44] Yang K., Dilthey B.G., Gross R.W. (2013). Identification and quantitation of fatty acid double bond positional isomers: a shotgun lipidomics approach using charge-switch derivatization. Anal. Chem..

[45] Ma X., Zhao X., Li J., Zhang W., Cheng J.-X., Ouyang Z. (2016). Photochemical tagging for quantitation of unsaturated fatty acids by mass spectrometry. Anal. Chem..

[46] Ichi I., Kono N., Arita Y., Haga S., Arisawa K., Yamano M. (2014). Identification of genes and pathways involved in the synthesis of Mead acid (20:3n−9), an indicator of essential fatty acid deficiency. Biochim. Biophys. Acta.

[47] Guillou H., Zadravec D., Martin P.G.P., Jacobsson A. (2010). The key roles of elongases and desaturases in mammalian fatty acid metabolism: insights from transgenic mice. Prog. Lipid. Res..

[48] Menzel J.P., Young R.S.E., Benfield A.H., Scott J.S., Wongsomboon P., Cudlman L., Cvačka J., Butler L.M., Henriques S.T., Poad B.L.J. (2023). Ozone-enabled fatty acid discovery reveals unexpected diversity in the human lipidome. Nat. Commun..

[49] Barnathan G. (2009). Non-methylene-interrupted fatty acids from marine invertebrates: occurrence, characterization and biological properties. Biochimie.

