# Supplementary material for: Alternative fatty acid desaturation pathways revealed by deep profiling of total fatty acids in RAW 264.7 cell line
Source: J Lipid Res. 2023 Jul 10;64(8):100410. doi: 10.1016/j.jlr.2023.100410 (PMC10407907; doi:10.1016/j.jlr.2023.100410)
Supplement: Supplemental information [file mmc2.pdf]

Supporting information for

Alternative fatty acid desaturation pathways revealed by deep profiling of total fatty acids in RAW264.7 cell line

Tian Xia[1], Xue Jin[2], Donghui Zhang[3], Jitong Wang[1], Ruijun Jian[1], Hang Yin[2,4,5], Yu Xia[1*]

[1] MOE Key Laboratory of Bioorganic Phosphorus Chemistry & Chemical Biology, Department of Chemistry, Tsinghua University, Beijing 100084, China

[2]School of Pharmaceutical Sciences, Tsinghua University, Beijing 100084, China

[3]State Key Laboratory of Precision Measurement Technology and Instruments, Tsinghua University, Department of Precision Instrument, Beijing 100084, China

[4]Tsinghua-Peking Center for Life Sciences, Tsinghua University, Beijing, 100084, China.

[5]Beijing Frontier Research Center for Biological Structure, Tsinghua University, Beijing, 100084, China.

To whom correspondence should be addressed:

Prof. Yu Xia, xiayu@mail.tsinghua.edu.cn

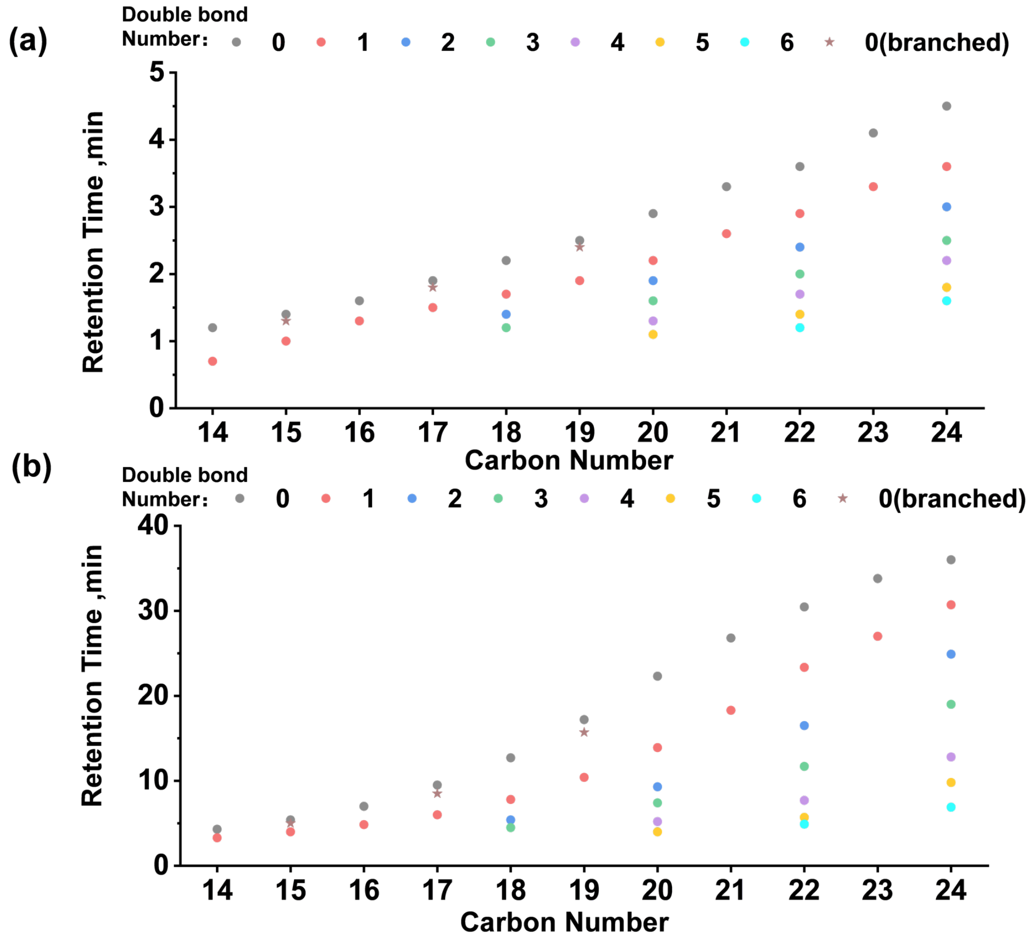

Supplemental Fig. S1 Retention time (RT) plot of AMPP-derivatized total fatty acid against the number of carbon atoms (14−24) and the number of double bonds (0−6) under (a) 7 min and (b) 50 min chromatographic separation.

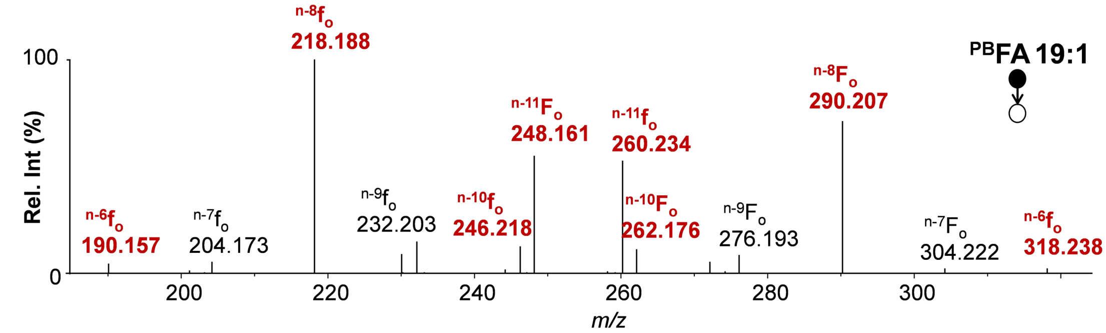

Supplemental Fig. S2. MS^2 CID spectrum of 2-acpy modified FA 19:1 in RAW 264.7 cells.

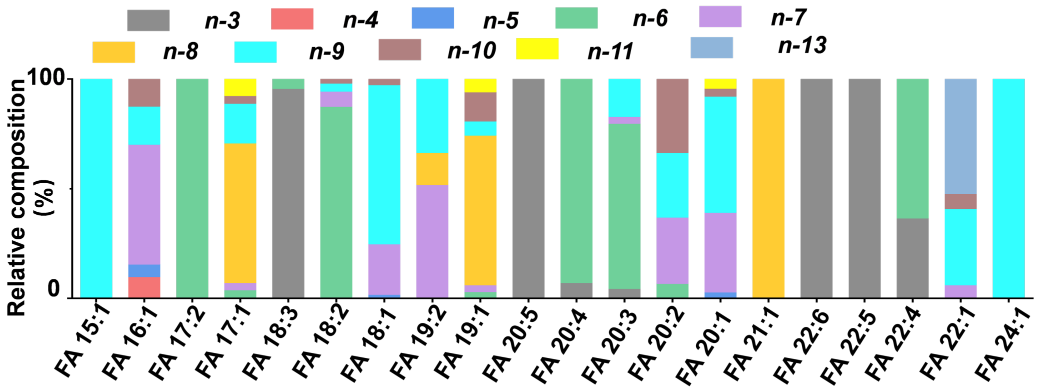

Supplemental Fig. S3. Relative compositions (%) of C=C location isomers in 20 groups of FAs in cell culture medium.

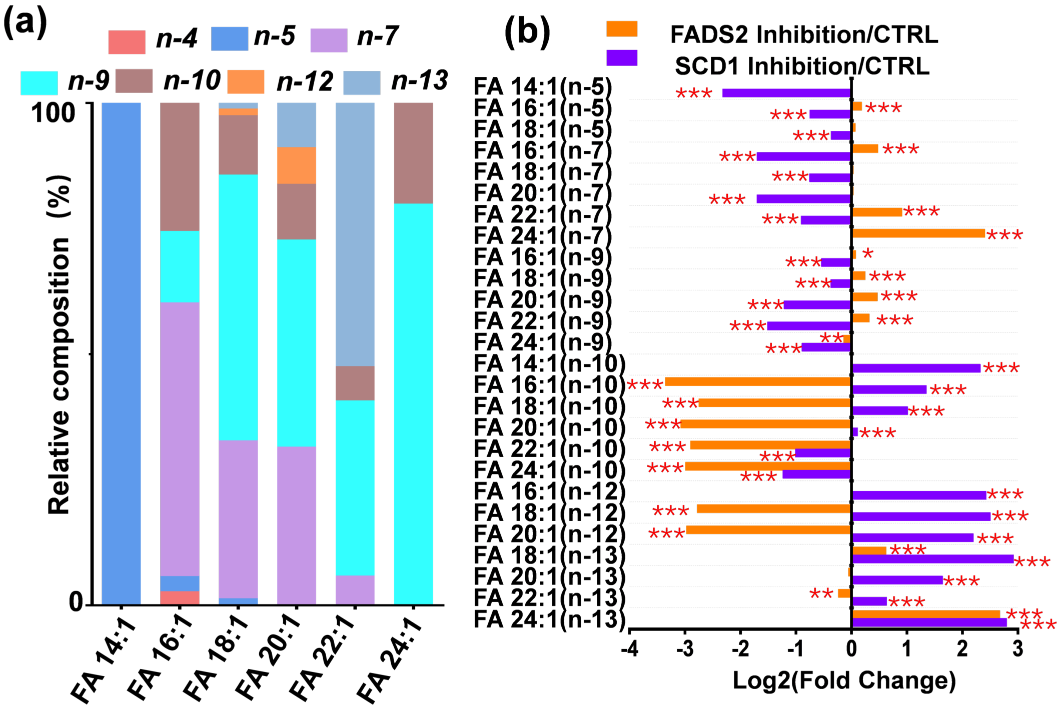

Supplemental Fig. S4. (a) Relative compositions (%) of C=C location isomers in even-chain monounsaturated fatty acid (MUFA) in RAW 264.7 cells. (b) Fold changes in MUFA levels induced by SCD1 inhibition or the FADS2 inhibition relative to the control measured in the RAW 264.7 cell lipidomes. N =6 biologically independent replicates. Differences between the two groups of samples were evaluated for statistical significance using the two-tailed student's t-test (*P < 0.05, **P < 0.01, ***P < 0.001)

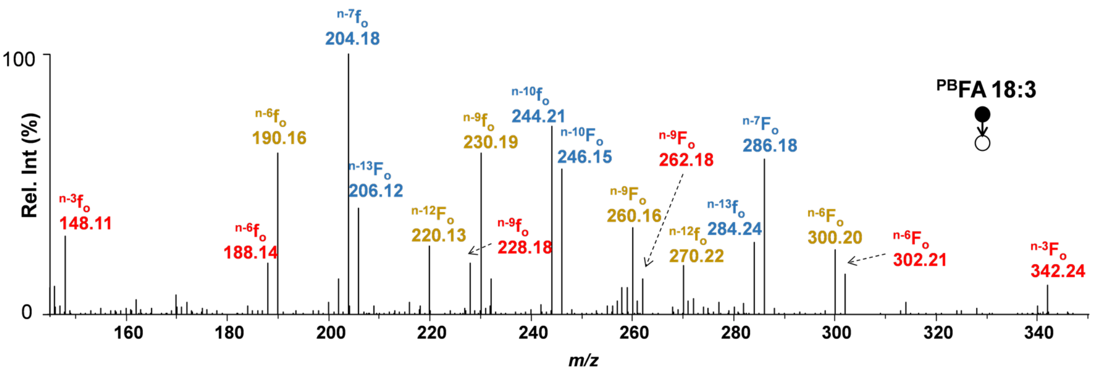

Supplemental Fig. S5. MS^2 CID spectrum of 2-acpy modified FA 18:3 in RAW 264.7 cells.

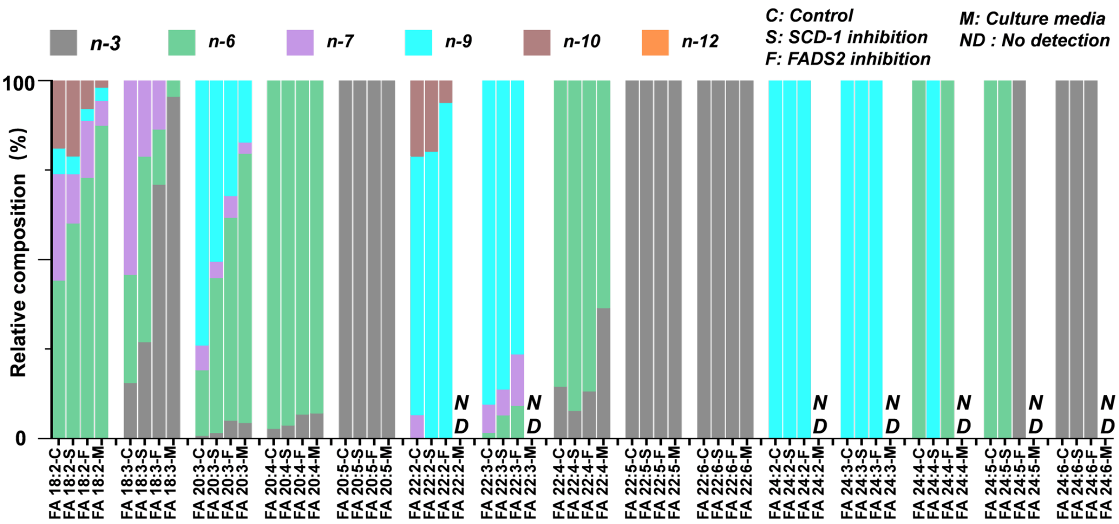

Supplemental Fig. S6. Comparison of %relative compositions of polyunsaturated fatty acid (PUFA) C=C location isomers in the culture medium, RAW 264.7 cells with and without inhibition of SCD1 or the FADS2 (N=6).
